# Detection of potentially inappropriate prescribing in the very old: cross-sectional analysis of the data from the BELFRAIL observational cohort study

**DOI:** 10.1186/s12877-015-0149-2

**Published:** 2015-12-02

**Authors:** Olivia Dalleur, Benoit Boland, Audrey De Groot, Bert Vaes, Pauline Boeckxstaens, Majda Azermai, Dominique Wouters, Jean-Marie Degryse, Anne Spinewine

**Affiliations:** Pharmacy department, Cliniques universitaires Saint-Luc, Université catholique de Louvain, Brussels, Belgium; Louvain Drug Research Institute (LDRI), Université catholique de Louvain, Brussels, Belgium; Geriatric Medicine, Cliniques universitaires Saint-Luc, Université catholique de Louvain, Brussels, Belgium; Ecole de Pharmacie, Université catholique de Louvain, and Centre Hospitalier Régional de Namur, Namur, Belgium; Institute of Health and Society Université catholique de Louvain, Brussels, Belgium; Department of Public Health and Primary Care, Katholieke Universiteit Leuven, Leuven, Belgium; Department of Family Medicine and Primary Health Care, Ghent University, Ghent, Belgium; Heymans Institute of Pharmacology, Ghent University, Ghent, Belgium; Pharmacy department, CHU Dinant-Godinne UCL Namur, Université catholique de Louvain, Yvoir, Belgium

**Keywords:** Inappropriate Prescribing, Aged 80 and older, Primary care, General practice, STOPP&START, Beers

## Abstract

**Background:**

Little is known about the prevalence and clinical importance of potentially inappropriate prescribing instances (PIPs) in the very old (>80 years). The main objective was to describe the prevalence of PIPs according to START (Screening Tool to Alert doctors to Right Treatment; omissions) and,STOPP (Screening Tool of Older Person’s Prescriptions; over/misuse) and the Beers list (over/misuse). Secondary objectives were to identify determinants if PIPs and to assess the clinical importance to modify the treatment in case of PIPs.

**Methods:**

Cross-sectional analysis of baseline data of the BELFRAIL cohort, which included 567 Belgian patients aged 80 and older in primary care. Two independent researchers applied the screening tools to the study population to detect PIPs. Next, a multidisciplinary panel of experts rated the clinical importance of the PIPs on a subsample of 50 patients.

**Results:**

In this very old population (median age 84 years, 63 % female), the screening detected START-PIPs in 59 % of patients, STOPP-PIPs in 41 % and Beers-PIPs in 32 %. Assessment of the clinical importance revealed that the most frequent PIPs were of moderate or major importance. In 28 % of the subsample, the relevance of the PIP was challenged by the global medical, functional and social background of the patient hence the validity of some criteria was questioned.

**Conclusion:**

Potentially inappropriate prescribing is highly prevalent in the very old. A good understanding of the patients’ medical, functional and social context is crucial to assess the actual appropriateness of drug treatment.

## Background

Potentially inappropriate prescribing (PIP) is highly prevalent in older adults and has been associated with adverse drug events, hospitalization and death [1–4]. A review reported that the median rate of inappropriate prescribing in primary care was around 20 % in patients aged over 65 years old [5]. But little is known about the prevalence of inappropriate prescribing in the very old, yet the latter represent a challenge for healthcare, because of multiple comorbidities, polypharmacy, frailty features and increased sensitivity to adverse drug events.

Several approaches exist to detect and reduce the burden of inappropriate prescribing in elderly, including the use of criterion-based screening tools, also called explicit tools [2, 6]. The most studied explicit tool is the Beers list, which was first published in 1991 [7] and last updated in 2012 [8]. In recent years, another explicit tool, the Screening Tool of Older Person’s Prescriptions (STOPP) and Screening Tool to Alert doctors to Right Treatment (START) [9], has been increasingly used [1]. The tool aims at detecting PIP in patients aged over 65 years old, which was the population target of most of the published studies using this tool. While Beers and STOPP address over- and misuse of inappropriate medications, the START tool allows for the detection of potentially inappropriate drug omissions. Some overlap of content between the STOPP and the 2012 Beers criteria has been described [10–13]. In the very old, to the best of our knowledge, no comparison with the updated 2012 Beers list has been performed. A recent study including inpatients aged 85 years and over measured the prevalence of PIPs according to STOPP&START and to a previous version of the Beers criteria (2003) [13]. This study showed a high prevalence of PIPs in that very old population (STOPP: 63 %; START: 54 %, Beers: 47 %) [13]. Moreover, the actual clinical relevance of those tools in the very old is unknown. Indeed, previous studies showed that potentially inappropriate prescribing detected by screening tools might differ from actually inappropriate prescribing [14–16].

The primary objective of this study was to determine the prevalence of PIP in community-dwelling patients aged 80 and older (the BELFRAIL population) [17] according to START (START-PIP), STOPP (STOPP-PIP) and Beers (Beers-PIP) tools.

Secondary objectives included the identification of determinants of PIPs in this population, and assessment of the clinical importance of a subsample of PIPs.

## Methods

### Study design, setting and participants

We performed a cross-sectional analysis of the baseline data of the BELFRAIL cohort (BF_C80+_) [17]. The BELFRAIL study is a prospective, observational, population-based cohort study of Belgian subjects aged 80 years and older [17]. The subjects were recruited in Belgium by their general practitioners (GPs) between November 2, 2008 and September 15, 2009, as described elsewhere [17]. This cohort excluded patients with severe dementia (mini mental state examination [18] MMSE <15/30), treated in palliative care and or as medical emergency. The protocol of this study was approved by the Biomedical Ethics Committee of the Medical School of the Université catholique de Louvain (UCL) of Brussels, Belgium (B40320084685). All patients gave written informed consent. Because patients with severe dementia (MMSE < 15) were excluded, all patients were able to give the informed consent themselves.

### Data collection

#### Medical and background data

For the 567 patients of the cohort, the GPs performed a detailed medical history and clinical examination [17]. The GPs reported the complete problem list of their patients as free text. Additionally, a structured questionnaire assessed the presence of 22 chronic conditions. Two researchers coded independently all the comorbidities listed by the GPs (OD and PB). Discrepancies were discussed with a third researcher (BV) until a consensus was reached. The Cumulative Illness Rating Scale (CIRS) was calculated [19–21]. The CIRS counts the number of 14 body systems affected with moderate disability, morbidity or extremely severe disease [19–21]. (Score varies from 0 to 14). Background data collection also included: cognitive impairment measured by the MMSE (score <25 was considered as “cognitive impairment”) [18], geriatric depression scale score GDS-15 (score >4 was considered as “possible depression”) [22], Tinetti fall risk score (score >24 was considered as “low fall risk”) [23], functional status according to the activities of daily living (ADL) score (ADL ranged between 6 and 30, lower score is related to functional dependency) [24], incontinence (reported by the GP), body mass index, familial status, and place of residence.

#### Drugs and inappropriate prescribing

GPs were asked to list the drugs the patient was taking on a regular basis or as needed. Drugs were coded and classified according to the Anatomical Therapeutic Chemical (ATC) classification system by one researcher (MA) [25].

Two researchers (OD and AD) independently retrospectively applied the 87 criteria of the STOPP&START tool [9] and the 3 categories of Beers’ criteria (drugs to avoid, to avoid regarding certain conditions/diseases, and to use with caution) [8] to the coded medical conditions and drugs. Discrepancies were discussed until consensus. For the analysis of the secondary outcomes, the Beers drugs to use with caution were not considered.

#### Determinants of PIP

Potential determinants of PIPs were tested among the following variables: medical (CIRS categorized as <4, or =4, or >4), geriatric (polypharmacy [≥5 drugs/day], MMSE [>25 (no cognitive impairment) or 20–24 or <20], ADL adjusted for gender [lowest quintile (higher dependence) or higher quintiles], GDS-15 [< or ≥5 (possible depression)], Tinetti score [25–28 (lowest fall risk) or 19–24 or ≤18], incontinence, body mass index [< or ≥21 kg/m^2^] [26, 27]) and social (age, gender, institutionalisation).

#### Clinical importance

On a randomly selected subsample of 50 patients, an expert panel (a general practitioner (JD), a geriatrician (BB) and a clinical pharmacist (AS), all with research experience in the field) was asked to independently rate the actual clinical importance of the recommendations (i.e., to add the drugs suggested by START to the treatment, or to discontinue the drugs detected by STOPP or Beers). Recommendations were classified following a previously defined method as minor, moderate, major, extreme, deleterious or not applicable (description of classifications can be found in Table 4) [14, 28]. Consensus on the clinical importance was reached when 2 experts agreed. To assess PIPs in the thorough context of the patient, the panel had access to the full record provided by the GP, and not only the coded conditions.

### Statistical analysis

Normally distributed continuous variables were expressed as mean ± standard deviation, while not normally distributed ones were summarized using the median and the inter-quartile range [Q25;Q75]. For categorical variables, numbers and percentages were presented. A univariate analysis and a multivariate logistic regression analysis were used to identify determinants of potentially inappropriate prescribing according to each tool. Variables with a p value of <0.05 in the univariate analysis were submitted for multivariate regression analysis. A *p* value <0.05 was considered statistically significant in the multivariate analysis. Statistical analyses were performed using IBM SPSS Statistics 20 (SPSS Inc., Chicago, IL, USA).

## Results

The characteristics of the 567 patients included at baseline in the cohort are presented in Table 1. Patients had a median age of 84 years, 63 % were female and they lived mainly at home (90 %). The most frequent comorbidities they presented were: hypertension (70 %), osteoarthritis (57 %) and ischemic disease (37 %). Eighty-one percent of the patients had at least one PIP in their medications: 59 % had START-PIPs (drug omissions), 41 % had STOPP-PIPs and 32 % had Beers-PIPs (drug overuse and/or misuse).

**Table 1:** Characteristics of the patients of the BELFRAIL cohort (*N* = 567)

**Characteristics of the patients**		
	Age (years), median [Q25;Q75]	84.0 [81.7;86.6]
	Gender, women, n (%)	356 (62.8)
	Resident in a nursing home, n (%)	57 (10.1)
	Number of drugs/day, median [Q25;Q75]	5 [4;7]
**Geriatric features**		
	Polypharmacy (≥ 5 drugs/day), n (%)	337 (61)
	ADL,^a^ median [Q25;Q75]	25 [21;27]
	Living alone at home, n (%)	212 (37.4)
	Urinary incontinence, n (%)	126 (22.2)
	Cognitive impairement, n (%)	89 (15.7)
	BMI < 21 kg/m², n (%)	49 (8.6)
	MMSE,^b^ median [Q25;Q75]	28 [25;29]
	Tinetti score,^c^ median [Q25;Q75]	27 [24;28]
	GDS-15,^d^ median [Q25;Q75]	2 [1;4]
	CIRS median [Q25;Q75]	4 [3;5]
**Most frequent comorbidities**		
	Hypertension, n (%)	396 (69.8)
	Osteoarthritis, n (%)	324 (57.1)
	Ischemic disease, n (%)	210 (37.0)
	Chronic heart failure, n (%)	166 (29.3)
	Chronic renal disease (GFR < 50 ml/min), n (%)	143 (25.2)
	Osteoporosis, n (%)	125 (22.0)
	Diabetes, n (%)	107 (18.9)
	Depression, n (%)	74 (13.1)
	COPD, n (%)	65 (11.5)
	Atrial fibrillation, n (%)	58 (10.2)
**Most frequent drugs prescribed, n patients (%)**		
	Antithrombotic agents (B01)	312 (55.0)
	Beta-blocking agents (C07)	238 (42.0)
	Agents acting on the renin-angiotensin system (C09)	237 (41.8)
	Psycholeptics (N05)	220 (38.8)
	Diuretics (C03)	189 (33.3)
	Lipid Modifying Agents (C10)	180 (31.7)
	Drugs for acid related disorders (A02)	138 (24.3)
	Calcium Channel Blockers (C08)	135 (23.8)
	Psychoanaleptics (N06)	131 (23.1)
	Cardiac Therapy (C01)	115 (20.3)
**Potentially inappropriate prescribing, n patients (%)**		
	START-PIPs	336 (59.3)
	STOPP-PIPs	232 (40.9)
	Beers-PIPs	180 (31.7)

*Abbreviations: ADL* activities of daily living, *BMI* body mass index, *CIRS* cumulative illness rating scale, *COPD* chronic obstructive pulmonary disease, *GDS* geriatric depression scale, *GFR* glomerular filtration rate, *MMSE* mini mental state examination, *PIPs* potentially inappropriate prescribing

^a^ADL ranged between 6 and 30, lower score is related to functional dependency

^b^MMSE <25 was considered as “cognitive impairment”

^c^Tinetti score >24 was considered as “low fall risk”

^d^GDS-15 score >4 was considered as “possible depression”

### Inappropriate prescribing

Overall, we found 1.13 ± 1.34 START-PIP per patient; range 0–8. In the 59 % of patients having at least one START-PIP, the average of START-PIPs rose to 1.90 ± 1.25 per affected patient.

Patients had on average 0.58 ± 0.92 STOPP-PIP in their list of prescriptions; range 0–10. The 41 % of affected patients had 1.43 ± 0.95 STOPP-PIP in their treatment.

The application of the Beers tool pointed out Beers-PIPs as drugs to avoid or to avoid in the presence of certain conditions in 32 % of the patients. The mean number of Beers-PIP in the treatment was 0.44 ± 0.79 per patient; range 0–6. In patients having at least one Beers-PIP, the average was 1.38 ± 0.80 per affected patient. In addition, Beers drugs that are labelled to be used with caution were found in 45 % of the patients.

Overall, 108 patients out of the 567 (19 %) had no PIP at all when considering START, STOPP and Beers tools. The most frequent PIPs are presented in Table 2. As far as underuse was concerned, the most frequent drug category using START was cardio-vascular (antiplatelet, statin, angiotensin-converting-enzyme inhibitors). The most frequent drug categories related to misuse or overuse were cardiovascular and psychotropic drugs (aspirin, benzodiazepines) and similar using STOPP and Beers. The prevalence of PIPs related benzodiazepine use with history of falls was less than 1 % (one patient). However, 19 % of the patients on benzodiazepines were at high fall risk according to their Tinetti score, and could therefore be assimilated to patients having PIPs.

**Table 2:** Most frequent potentially inappropriate prescribing events according to START, STOPP and/or Beers criteria

	Therapeutic class/medication ± disease	Prevalence % (n)
**Under prescribing according to START**		
	Aspirin or clopidogrel with a documented history of atherosclerotic coronary, cerebral or peripheral vascular disease in patients with sinus rhythm	15,0 (85)
	Calcium and vitamin D supplement in patients in the presence of known osteoporosis	13,9 (79)
	ACE inhibitor in the presence of chronic heart failure	12,7 (72)
	Statin therapy with a documented history of coronary, cerebral or peripheral vascular disease, where the patient’s functional status remains independent for activities of daily living and life expectancy is greater than 5 years	9,5 (54)
	Antiplatelet therapy in diabetes mellitus with coexisting major cardiovascular risk factors (hypertension, hypercholesterolemia, smoking history)	9,5 (54)
**Over/misprescribing according to STOPP or/and Beers**		
STOPP and Beers	Aspirin for primary cardiovascular prevention^a^	16,9 (96)
Beers	Nonbenzodiazepine (“Z”) hypnotics (i.e., eszoplicone, zaleplon, zolpidem)	6,2 (35)
STOPP	Any duplicate drug class prescription	6,2 (35)
Beers	Benzodiazepines in the presence of dementia and cognitive impairment	5,8 (33)
STOPP and Beers	Long-acting benzodiazepines	4,9 (28)
STOPP	Aspirin at dose > 150 mg/day	4,4 (25)
STOPP	NSAIDs with moderate to severe hypertension	3,7 (21)
Beers	Tertiary TCAs, alone or in combination	2,6 (15)

*Abbreviations: ACE* angiotensin-converting-enzyme, *NSAIDs* nonsteroidal anti-inflammatory drugs, *TCA* tricyclic antidepressant

^a^To be used with caution in adults >80 years old for primary prevention of cardiac events in Beers 2012; to be avoided in those with no history of coronary, cerebral, or peripheral vascular symptoms or occlusive events in STOPP

### Determinants of PIP

The results of the multivariate analysis are shown in Table 3. A lower comorbidity score was a determinant of lower odds of having START-PIPs (odds ratio [OR] 0.2, 95 % confidence interval [CI] 0.1–0.3 for CIRS <4 vs. >4). Functional dependence (lowest quintile of ADL, range in women 6–18, range in men 6–21) was the only determinant of having STOPP-PIP (OR 1.5, 95 % CI 1.0–2.4). Beers-PIPs were also associated with the CIRS (OR 0.4, 95 % CI 0.3–0.7 for CIRS <4 vs. >4, and OR 0.6, 95 % CI 0.4–0.9 for CIRS =4 vs. >4). Living in a nursing home was another determinant of Beers-PIPs (OR 1.8, 95 % CI 1.0–3.4). Other social and geriatric features were not related to PIPs.

**Table 3:** Determinants of potentially inappropriate prescribing in the study population (multivariate analysis)

	Covariates	OR [95 % CI]	*p* value
**START-PIP**			
	ADL lowest quintile^a^	0.8 [0.4–1.5]	0,523
	Age, per year	1.0 [0.9–1.1]	0,227
	CIRS >4	1.0	
	CIRS <4	0.2 [0.1–0.3]	<0,001
	CIRS=4	0.6 [0.3–1.1]	0,090
	GDS-15 >4^b^	1.2 [0.7–2.0]	0,442
	Gender, women	0.9 [0.6–1.4]	0,727
	Tinetti ≤ 18^c^	1.0	
	Tinetti 25–28	0.5 [0.2–1.2]	0,130
	Tinetti 19–24	0.9 [0.3–2.2]	0,840
**STOPP-PIP**			
	ADL lowest quintile	1.5 [1.0–2.4]	0.050
	Age, per year	1.0 [0.9–1.0]	0.957
	Gender, women	1.2 [0.9–1.8]	0.211
	Resident in a nursing home	1.8 [0.9–3.2]	0.056
**BEERS-PIP**			
	ADL lowest quintile	1.1 [0.7–1.9]	0.558
	Age, per year	0.9 [0.9–1.0]	0.515
	CIRS >4	1.0	
	CIRS < 4	0.4 [0.3–0.7]	<0.001
	CIRS = 4	0.6 [0.4–0.9]	0.041
	GDS-15 >4	1.5 [0.9–2.3]	0.094
	Gender, women	1.2 [0.8–1.8]	0.364
	Resident in a nursing home	1.8 [1.0–3.4]	0.045

Hosmer-Lemeshow goodness-of-fit *P*-value for START = 0.42; STOPP = 0.15; Beers = 0.89 indicating that the models are a good fit for the data

*Abbreviations: ADL* activities of daily living, *CI* confidence interval, *CIRS* cumulative illness rating scale, *GDS* geriatric depression scale, *OR* odds ratio, *PIPs* potentially inappropriate prescribing

^a^ADL lowest quintile: lower ADL score is related to functional dependency

^b^GDS-15 score >4 was considered as “possible depression”

^c^Tinetti score >24 was considered as “low fall risk”; ≤ 18 was “high fall risk”

### Clinical importance of the recommendations to modify the treatment in the presence of PIP

In the subsample of 50 patients, the experts examined 143 PIPs (i.e.,: 44 STOPP-PIPs, 65 START-PIPs and 34 Beers-PIPs [including 3 PIPs overlapping the STOPP-PIPs], concerning 17 different criteria of the START tool, 18 criteria from STOPP and 20 criteria from Beers). The experts agreed on the clinical importance of 83 % of the PIPs. The clinical importance of the same criterion could vary in different patients according to each patient’s individual context.

Forty-three PIPs cases (30 %) were rated of “major” importance, while 33 PIPs (23 %) were considered of moderate importance. The experts rated two PIPs as “minor”. One START-PIP appeared to be “deleterious”. Examples are provided in Table 4. Finally, the experts agreed that 40 PIPs (28 %) were “non-applicable” according to the individual context and even considered 19 of those cases (13 %) as actually appropriate prescribing.

**Table 4:** Clinical importance of potentially inappropriate prescribing criteria according to the expert panel

Examples			
Major clinical importance (*n* = 43)	*Modification of the treatment according to this criteria may prevent serious morbidity, including readmission, serious organ dysfunction, serious adverse drug event*		
		*Criterion*: START-PIP “Angiotensin converting enzyme (ACE) inhibitor with chronic heart failure”.	*Context*: The GP reports chronic heart failure, with marked limitation of physical activity and dyspnea, and a recent episode of congestive heart failure.
		*Criterion*: STOPP-PIP “Calcium channel blockers with NYHA class III or IV heart failure”/Beers- PIP “Diltiazem in heart failure”.	*Context*: The medical history and the clinical examination confirm that the patient has NYHA class III heart failure.
		*Criterion*: Beers- PIP “Anticholinergics in dementia and cognitive impairment”.	*Context*: The patient has cognitive impairment (MMSE = 22/30^a^) and takes several drugs with anticholinergic properties (amisulpride, trihexyfenidyl).
Moderate clinical importance (*n* = 33)	*Modification of the treatment according to this criteria brings care to a more acceptable and appropriate level of practice or that may prevent an adverse drug event of moderate importance*		
		*Criterion*: START-PIP “Statin therapy in diabetes mellitus if coexisting major cardiovascular risk factors present”.	*Context*: The patient is 87 years, and still has good cognitive and functional status. She has diabetes, hypertension and hypercholesterolemia.
		*Criterion*: STOPP-PIP “Long-term long-acting benzodiazepines”.	*Context*: The patient has been taking 8mg prazepam every day for more than a month. She has low fall risk (Tinetti score 26/28^b^) but she has cognitive impairment (MMSE=18/30).
		*Criterion*: STOPP-PIP/Beers-PIP “Aspirin in primary cardiovascular prevention”.	*Context*: The patient has no history of coronary, cerebral or peripheral vascular symptoms or occlusive event.
		*Criterion*: Beers- PIP “Tertiary tricyclic antidepressants”.	*Context*: The patient is on clomipramine for “depressive tendencies” according to the GP. The GDS-15 score is low (3/15^c^). Non pharmacologic or safer alternatives are available.
Minor clinical importance (*n* = 2)	*Modification of the treatment according to these criteria brings no benefit or minor benefit, depending on professional interpretation*		
		*Criterion:* Beers-PIP “Avoid antiarrhythmic drugs as first-line treatment of atrial fibrillation”.	*Context:* This patient receives amiodarone and does not suffer from any side effect of this drug.
		*Criterion:* Beers-PIP “Avoid long duration sulfonylurea”.	*Context:* The patient is on gliclazide extended release formula. He is intolerant to metformine. No hypoglycemia were reported.
Deleterious clinical importance (*n* = 1)	*Modification of the treatment according to this may lead to adverse outcome.*		
		*Criterion:* START-PIP “Statin therapy in diabetes mellitus if coexisting major cardiovascular risk factors present”*.*	*Context:* The patient has a documented allergy to statins.
Non applicable (*n* = 40)	*The criterion is not applicable to the individual context of the patient.*		
		*Criterion:* START-PIP “beta-blocker with chronic stable angina”.	*Context:* The patient had a single episode of suspected angina in the past, and he has asthma.
		*Criterion*: START-PIP “Aspirin therapy in diabetes mellitus if coexisting major cardiovascular risk factors present”.	*Context*: The patient is already on anti-vitamin K and he has no acute coronary disease.
		*Criterion:* STOPP-PIP “Any duplicate drug class prescription”.	*Context:* The prescription includes a patch of nitroglycerin and tablets of isosorbide dinitrate. However, in his notes, the GP specifies that the patient uses the tablets “as needed” only.
		*Criterion*: STOPP-PIP “Long-term use of NSAID for symptom relief of mild osteoarthritis”.	*Context*: The 83 year old patient has chronic knee pain despite the use of paracetamol. Unfortunately, his severe respiratory and cardiac status is a contra-indication to surgery and he is intolerant to alternatives to NSAID. He is on proton-pump inhibitor.
		*Criterion:* Beers*-*PIP “Avoid antipsychotics in dementia & cognitive impairment”.	*Context:* This patient has cognitive impairment but also a long story of psychiatric disorders.
		*Criterion:* Beers*-*PIP “Avoid benzodiazepines for the treatment of insomnia, agitation, or delirium”.	*Context:* This patient received alprazolam to improve her sleep in a context of severe chronic anxiety.

*Abbreviation: GDS-15* geriatric depression scale, *GP* general practitioner, *MMSE* mini mental state examination, *NSAID* non-steroidal anti-inflammatory drugs, *NYHA* New York Heart Association Functional Classification, *PIP* potentially inappropriate prescribing

^a^MMSE<25 was considered as “cognitive impairment”

^b^Tinetti score >24 was considered as “low fall risk”

^c^GDS-15 score >4 was considered as “possible depression”

The 40 “non applicable” cases could be divided into two categories. The first category encompassed 25 cases where the experts found nuances within the detailed full record of the patient, including the comprehensive geriatric assessment, which questioned the applicability of the criteria. In the second category (*n* = 15), the experts questioned the content validity of the criteria. Examples are provided in Table 5. Comparing the PIPs detected by the three tools, START-PIPs were the most frequently rated as “non applicable”.

**Table 5:** How a holistic approach of the patient challenges the PIPs detected by explicit screening tools

Elements of the patient’s record that influence the applicability of the criteria^a^
• Level of severity of a disease • Certainty of the diagnoses • Timing of the medical history (recent event vs. long ago) • Actual intake of the drug that differs from the prescription • Patient’s preferences and objectives • Mental status of the patient and associated psychiatric conditions • Absence of alternative treatment • Patient’s pain status • Drug-drug interactions • Risk factors for bleeding or for stroke • Contra-indication • Allergies
Situations that question the content validity of the criteria:
• START-PIP in patients already treated by suitable alternative medications e.g., “Proton pump inhibitor with severe gastroesophageal acid reflux disease” in a patient already on histamine H2-receptor antagonist. • START-PIP “Warfarin in the presence of chronic atrial fibrillation” in patients with low stroke risk • START-PIP “Regular inhaled β2-agonist or anticholinergic agent for mild-to-moderate asthma or COPD” in a patient with asthma due to acid reflux • STOPP-PIP “Any duplicate drug class prescription” because insufficiently defined. • Beers-PIPs mentioning that a medication should be avoided as “first-line therapy” because such a feature is often difficult to detect • Beers-PIP “Avoid antidepressants in dementia & cognitive impairment” in a patient with severe depression

^a^See examples in Table 4

## Discussion

### Summary

For the first time, this study described inappropriate prescribing and their determinants in a large representative sample of community-dwelling very old patients. In this population, the prevalence of PIPs was high. Potentially inappropriate omissions, detected by the START tool were more prevalent (59 % of the patients) than overuse of treatment (STOPP: 41 %; Beers’ drugs “to avoid”: 32 %). Drugs from the cardiovascular and neurologic systems were the most frequently involved in PIPs. Among medical, social and geriatric features, the CIRS was related to having START- and Beers-PIPs, place of residency was associated with Beers-PIPs and the functional dependence was determinant of having STOPP-PIPs.

The evaluation of the relevance of the criteria showed that a holistic approach of the patient changed the applicability of the criteria in 17 % of PIP cases. More importantly, in 10 % of cases, the recommendation to modify the drug regimen was not valid, and could even be considered as deleterious, which is not acceptable. Finally, the experts did not rate similarly the clinical importance of the criteria in 17 % of cases. This illustrates the subjectivity of the assessment of the patient’s context and the variable importance acknowledged to inappropriate prescribing according to the evaluator.

### Comparison with existing literature

The prevalence detected in our patients aged 80 and older (START : 59 %; STOPP: 41 %; Beers’ drugs “to avoid”: 32 %) did not substantially differ from the prevalence reported in the literature with populations including younger patients in whom the range of prevalence of START-PIP is 23 to 68 % [29–32], STOPP-PIP 18-60 % [30, 32–37], and Beers-PIP 12.5–42 % [38, 39]. This observation is consistent with a study published in 2015 that compared the prevalence of START-PIPs, STOPP-PIPs and Beers-PIPs in patients aged between 75 and 84 years vs patients aged 85 and over, in the hospital setting [13]. The prevalence of PIPs was similar in both groups [13]. It should be noted that prevalence of PIPs varies greatly from studies, depending of the setting and the range of the criteria of the tools that were taken into account.

In younger patients, STOPP-PIPS have been related to polypharmacy, age, institutionalisation, and increased comorbidity), while START-PIPs were variably related to age, female gender, and increased comorbidity [1, 13, 40]. Our results confirm the importance of the comorbidity burden in the risk of having PIPs.

Only a few previous studies looked at the clinical importance of PIPs detected by explicit tools in patients [14–16]. Our analysis on the subsample showed the same trends as previous studies on the Beers list [15, 16] and STOPP [14]: a substantial proportion of PIPs were actually appropriate, the relevance varied according to the drug type and some criteria appeared less controversial than others (Table 5). Our review of the subsample of patients reinforces the idea that PIPs are actually only potential and that solely looking at the criteria is not sufficient to decide if the prescribing is inappropriate or not. A holistic approach to the patient challenges the PIPs detected by explicit screening tools, as illustrated in Table 5.

### Strengths and limitations

This study goes one step further than previous observational studies on PIPs, by providing important findings about the validity of the use of explicit tools and highlighting how a holistic approach matters when reviewing the treatment.

This study presents some limitations. The data used to detect PIPs were not prospectively collected for the purpose of this analysis. Therefore, the quantity of PIPs might have been underestimated. For example, PIPs related to the history of falls, which were frequently reported in previous studies [41], were infrequent in our study (likely due to under-reporting of falls as a medical condition by GPs). However, we observed that a fifth of the patients on benzodiazepines were at high fall risk. Criteria related to delirium and dementia were expected to be infrequently encountered because these patients were excluded from the cohort.

The analysis on the clinical relevance of the criteria was only performed on a small subsample of PIPs. This assessment was designed to provide an insight but did not intend to comprehensively evaluate the content of the full criteria lists. Further studies should assess the actual inappropriateness of all the drugs listed on the tools in larger extent. However, this subsample allowed to discuss the most frequent PIPs and enabled us to identify several important points for discussion on the validity of the tools.

### Implications for research and/or practice

Based on this analysis, including the examples detailed in Table 5, we suggest some important modifications to the tools to improve their validity and applicability (Table 6). The main suggestion for the future use of screening tools in daily practice is that these tools can only be used in addition of an assessment of actual appropriateness of prescribing by clinicians with a good understanding of the patient global health situation and full access to the patient’s history. In many ways, the GP appears as the foremost potential user of the tools. Indeed, the GP knows the patient the best, thanks to a long relationship and global vision of the patient medical, social and functional status. Very recently, an updated version of the STOPP&START tool was published [42]. Some of the criteria for which the validity was questioned in our study have been removed. However, we believe that most recommendations in Table 6 remain valid. Moreover, the message about the paramount importance of using the tools as part of a holistic approach is applicable to any explicit criteria list.

**Table 6:** Recommendations to improve the validity and applicability of explicit tools

Recommendations to improve the validity of the criteria	Recommendations to improve the applicability of the criteria
• mention of contra-indications of the criteria • no contradictions between criteria • no overlap between criteria • precise range of application of the criteria (inclusion criteria) • mention of time to benefit [45, 46]	• clear definitions (conditions, diseases, drug categories) • monitoring tips • suggestions of alternatives (pharmacological and non-pharmacological) • mention of adaptation to functional and cognitive status, life-expectancy, and multimorbidity.

Our results therefore somewhat question the application of explicit screening tools to administrative databases. This approach, which was regularly performed in previous studies [34, 43], is valuable to have a global insight into PIPs patterns and the most frequently encountered drugs. But the prevalence and frequencies should be interpreted with caution. Issues identified can only be deemed potentially inappropriate owing to the limited clinical information available in such databases. Application of explicit tools to large databases should be refined so as to take into account factors that decrease applicability of some criteria (e.g., contra-indications or presence of alternative drugs for START criteria).

Perspectives for future research are provided by this baseline analysis of the BELFRAIL cohort. Longitudinal analysis should compare the incidence of geriatric adverse events (death, hospital admissions, falls, adverse drug events) and costs of care in patients having or not confirmed PIPs at baseline. Additional qualitative research could enlighten some of the barriers to implement screening tools. Furthermore, the viewpoint of the patient on the appropriateness of his own treatment should be explored.

## Conclusions

Our observations highlight the high prevalence of PIPs in very old patients in primary care. The medication review should be part of a comprehensive process to optimize pharmacotherapy. Explicit tools help to revise the treatment but will never replace good clinical judgement [44]. The general practitioner plays a key-role in the management of chronic drug treatment and is therefore potentially in the best position to collaborate and to apply the explicit tool. A good understanding of the patients’ medical, functional and social context is crucial to assess the actual appropriateness of drug treatment.
